# Prognostic model development using novel genetic signature associated with adenosine metabolism and immune status for patients with hepatocellular carcinoma

**DOI:** 10.1007/s13105-024-01061-8

**Published:** 2024-11-15

**Authors:** Yidan Chen, Kemei Wang, Xingyun Zhang, Dongying Tao, Yulong Shang, Ping Wang, Qiang Li, Yansheng Liu

**Affiliations:** 1 National Clinical Research Center for Digestive Diseases and Xijing Hospital of Digestive Diseases, Xijing Hospital, Air Force Medical University, Xi’an, China; 2 School of Basic Medicine, Air Force Medical University, Xi’an, China; 3 Department of General Medicine, Xijing Hospital, Air Force Medical University, Xi’an, China; 4 Department of Pediatric, Xijing Hospital, Air Force Medical University, Xi’an, China; 5 Department of Gastroenterology, Dongying People’s Hospital, Dongying, China

**Keywords:** Hepatocellular carcinoma, Adenosine metabolism, Immune status, Genetic signature, Immune checkpoint

## Abstract

The high mortality rate of hepatocellular carcinoma (HCC) is partly due to advanced diagnosis, emphasizing the need for effective predictive tools in HCC treatment. The aim of this study is to propose a novel prognostic model for HCC based on adenosine metabolizing genes and explore the potential relationship between them. Regression analysis was performed to identify differentially expressed genes associated with adenosine metabolism in HCC patients using RNA sequencing data obtained from a public database. Adenosine metabolism-related risk score (AMrisk) was derived using the least absolute shrinkage and selection operator (LASSO) Cox regression and verified using another database. Changes in adenosine metabolism in HCC were analyzed using functional enrichment analysis and multiple immune scores. The gene expression levels in patient samples were validated using quantitative reverse transcription polymerase chain reaction. Thirty adenosine metabolism-related differentially expressed genes were identified in HCC, and six genes (ADA, P2RY4, P2RY6, RPIA, SLC6A3, and VEGFA) were used to calculate the AMrisk score; the higher the risk scores, the lower the overall survival. Moreover, immune infiltration activation and immune checkpoints were considerably higher in the high-risk group. Additional in vitro experiments validated the enhanced expression of these six genes in HCC. The established predictive model demonstrated that adenosine metabolism-related genes was significantly associated with prognosis in HCC patients.

**Supplementary Information:**

The online version contains supplementary material available at 10.1007/s13105-024-01061-8.

## Key Points

- The establishment of a prognostic gene marker for HCC was achieved based on the analysis of adenosine metabolism-related genes. The stability of the prognostic model was validated through external verification. Explored the immune related mechanisms of HCC and analyzed drug sensitivity.

**Supplementary Information:**

The online version contains supplementary material available at 10.1007/s13105-024-01061-8.

## Introduction

According to the 2021 World Health Organization report, primary liver cancer ranks as the sixth most prevalent global malignancy and stands as the third leading cause of mortality, with an annual incidence of 841,000 and 782,000 deaths, approximately [1]. Although surgery is the mainstay of treatment for hepatocellular carcinomas (HCC), the recurrence rate after surgical resection remains high. Owing to poor prognosis, limited treatment options, and the high rate of recurrence and metastasis, treatment of unresectable HCC remains challenging [2, 3]. Despite recent advancements in treatment methods, the overall survival (OS) of HCC patients remains low, which could be attributed to the large heterogeneity of HCC and the challenges associated with traditional histopathological classification in accurately assessing the prognosis and providing more precise treatment [4–6]. Therefore, developing predictive models for strategizing treatment plans and assessing prognosis is imperative to improving clinical outcomes in HCC patients.

Altered metabolic status is a hallmark characteristic of cancer cells, and the metabolic processes of cancer cells across cancer types and genetic backgrounds are universally dependent on the synthesis and use of nucleotides. Adenosine is a key component of nucleotides that is mainly involved in cellular energy conversion processes and various signal transduction pathways [7]. Differential expressions of key genes are involved in adenosine metabolism in tumor patients. Furthermore, adenosine metabolism-related signaling pathways may serve as primary regulators of antitumor immunity within the tumor microenvironment (TME), directly influencing cancer cell survival, proliferation, and metastasis [8–10]. Adenosine accumulation is implicated in tumor immune escape, aggressive proliferation, and metastasis [11–13]. Although studies on adenosine metabolism and the mechanisms of HCC development focused on changes in adenosine metabolic pathways during cancer progression, the potential association between adenosine metabolism and the clinical progression of HCC remains unknown.

Tumor growth is determined not only by genetic alterations in malignant cells but also by the TME [14, 15]. Cancer cells and host immune responses interact in the TME to promote or inhibit cancer progression. Schreiber et al. originally proposed that few cancer cells are capable of evading immune system recognition through various mechanisms and succeed in achieving “immune escape” [16]. Given that tumor cells in HCC achieve immune escape through immune checkpoints [17–19], immunotherapy with immune checkpoint inhibitors (ICIs) has become a new therapeutic strategy for HCC patients. ICIs target anti-programmed cell death receptor 1 (PD-1) and programmed cell death receptor ligand 1 (PD-L1), alleviating anti-tumor immunity suppression thereby enhancing the body’s ability to fight cancer [20, 21]. Many other assay targets, such as cytotoxic T-lymphocyte-associated protein 4 (CTLA-4) and lymphocyte-activated gene-3 (LAD-3), have entered the clinical period and have reported promising results [22–24]. These studies highlight that immune checkpoint blockade immunotherapy and related combination therapies are the emerging trends for treating advanced HCC.

In this study, the HCC cohort from The Cancer Genome Atlas (TCGA) was used as the training set to select adenosine metabolism-related differentially expressed genes (AMRDEGs) to establish the adenosine metabolism-related risk score (AMrisk). Subsequently, the cohort data from the International Cancer Genome Consortium (ICGC) database was used to verify the stability of AMrisk. The prognostic nomogram was developed by integrating AMrisk with clinical features, and the association and functional analysis between AMrisk and TME were evaluated. Furthermore, the expression of AMRDEGs was verified using in vitro experiments. In summary, our study incorporated the biological characteristics of adenosine metabolism into a predictive model and established a new prognostic risk model for HCC, providing valuable insights for formulating treatment strategies and improving treatment efficacy.

## Materials and methods

### Data and resources

RNA sequencing data and the clinical characteristics of HCC patients were obtained from TCGA database (https://portal.gdc.cancer.gov/). The RNA expression profiles included 406 HCC samples and 58 paracancerous normal tissue samples. Information on 403 groups of HCC samples with corresponding clinical characteristics was used for subsequent analyses. The RNA sequencing data of 260 HCC patients and their corresponding clinical features were obtained from the ICGC database (https://dcc.icgc.org/). Human genes related to adenosine metabolism were obtained from a study by Yu et al. [25].

### Identification and validation of adenosine metabolism-related prognostic genes

AMRDEGs between tumor and normal samples in TCGA dataset were searched by using “limma” R package. The screening thresholds in the R package algorithm were set as p-values < 0.05 and |log2(fold change)| > 1 [26]. Univariate Cox regression analysis was used to determine the AMRDEGs related to prognosis (p-value < 0.05 indicated statistical significance).

### Establishment and validation of AMrisk

The least absolute shrinkage and selection operator (LASSO)-Cox regression analysis is a widely employed approach for constructing prognostic models that provide both satisfactory prediction accuracy and interpretability [27]. Therefore, LASSO-Cox regression model analysis using the “glmnet” R package in conjunction with the “survival” package analysis was used in this study. The LASSO-Cox weighting coefficient used the expression level of a single gene to establish AMrisk as follows: AMrisk= ∑ (expression of genex × coefficient of genex). Based on the median AMrisk values, HCC patients were stratified into low- and high AMrisk groups. The Kaplan-Meier method was employed to analyze survival status, and the log-rank test was utilized for inter-group comparisons of survival curves. To determine the predictive performance for 1, 3, and 5-year survival outcomes and the specificity and sensitivity of AMrisk, receiver operating characteristic (ROC) analysis was performed using the “timeROC” R package, and the areas under the curve (AUC) were computed accordingly. Furthermore, the applicability of AMrisk to the ICGC dataset was validated using an identical methodology.

### Construction and validation of prognostic column-line diagrams

Univariate and multivariate Cox analyses were performed to investigate the association between clinical characteristics (age, sex, grade [TCGA], and stage) and prognosis in HCC patients. Additionally, the independent and superior predictive abilities of AMrisk gene markers were assessed (the criterion for statistical significance was set at a level of *P* < 0.05). Hazard ratios (HRs) and corresponding 95% confidence intervals (CIs) were calculated for each variable. After conducting collinearity testing, a nomogram was developed using the “rms”, ‘survival’, and “regplot” packages in R software to predict the 1-, 3-, and 5-year survival rates in patients with HCC, incorporating patient clinical characteristics and AMrisk. Subsequently, a calibration curve was constructed to evaluate the concordance between the observed and predicted survival outcomes; closer alignment with the ideal 45° line indicated improved predictive performance of the nomogram model. Finally, time-dependent ROC curve analysis was employed to assess the prognostic accuracy of the nomogram at 1, 2, and 3 years.

### Functional enrichment analysis

The potential biological functions of the differentially expressed genes (DEGs) between the low- and high-risk groups were investigated using Gene Ontology (GO) [28] enrichment analysis, as well as Kyoto Encyclopedia of Genes and Genomes (KEGG) [29] enrichment analysis. The results were visualized by using the “clusterProfiler,” “org.Hs.eg.db,” “enrichplot,” and “ggplot2” R packages. The GO enrichment analysis was divided into three parts: biological processes, molecular functions, and cellular components.

### TME and immune response analysis

The analysis of changes in the infiltration fraction of 16 immune cell subpopulations and the activity of 13 immune-related pathways in the high- and low-risk groups involved utilizing R packages “GSVA,” “limma,” and “GSEABase” for single-sample gene set enrichment analysis (ssGSEA) calculations. The limma, ESTIMATE, reshape2, and ggpubr R packages were used for Estimation of Stromal and Immune cells in MAlignant Tumor tissues using Expression data (ESTIMATE) analysis, and to compare the levels of immune cell and interstitial cell infiltration between the high- and low-risk groups.

### Cell lines culture

Human normal liver tissue cells L-02 and liver cancer cells SMMC-7721, Hep-G2, Hep-3B, and Huh-7 were obtained from the Stem Cell Bank of the Chinese Academy of Sciences, China Cell Line Resource Infrastructure, and China Hepatocellular Carcinoma Cell Resource Infrastructure. L-02 and SMMC-7721 cell lines were cultured in Roswell Park Memorial Institute (RPMI) 1640 medium supplemented with 10% high-quality fetal bovine serum (originally sourced from South America; ic − 1905; BioCytoSci, TX, USA) and 1% penicillin-streptomycin. The Hep-G2, Hep-3B, and Huh-7 cell lines were cultured in a modified variant of Dulbecco’s modified Eagle’s medium (DMEM) that consisted of 10% fetal bovine serum and 1% penicillin-streptomycin. All cells were cultured in a CO2 incubator with a temperature of 37 °C and a controlled concentration of 5%.

### RNA extraction and quantitative reverse transcription PCR (qRT-PCR)

The TRIzol reagent (Invitrogen, USA) was utilized to extract RNA from the cell lines according to the manufacturer’s instructions. The Advantage RT-PCR kit (Takara Bio) was employed for reverse transcription. Subsequently, a CFX96™ Real-Time PCR detection system (Bio-Rad Laboratories, Hercules, CA, USA) in combination with the SYBR Premix Pro Taq HS qPCR kit (Accurate Biotechnology, Hunan, China) was used for RT-PCR analysis. The relative expression levels were determined using the 2 ^−ΔΔCt^ method. Please refer to Online Resource 1 for primer sequences.

### Statistical analyses

R software (version 4.3.1) was utilized for conducting statistical analyses and generating visualizations. The Kolmogorov-Smirnov test was employed to assess the normality of the distribution. Data that followed a normal distribution were presented as mean ± standard error of the mean. Student ‘s t-test was used for two-group comparisons, and the Student–Newman–Keul test after one-way ANOVA was used for multiple-group comparisons. To compare three or more groups with data that does not follow a normal distribution, the Kruskal-Wallis test was employed. Kaplan–Meier survival curve analysis and log-rank tests were used to analyze OS. Spearman’s rank correlation was used to test the association between AMrisk and immune checkpoint expression levels. All statistical tests were two-sided, and P < 0.05 was considered statistically significant.

## Results

**Fig. 1:**
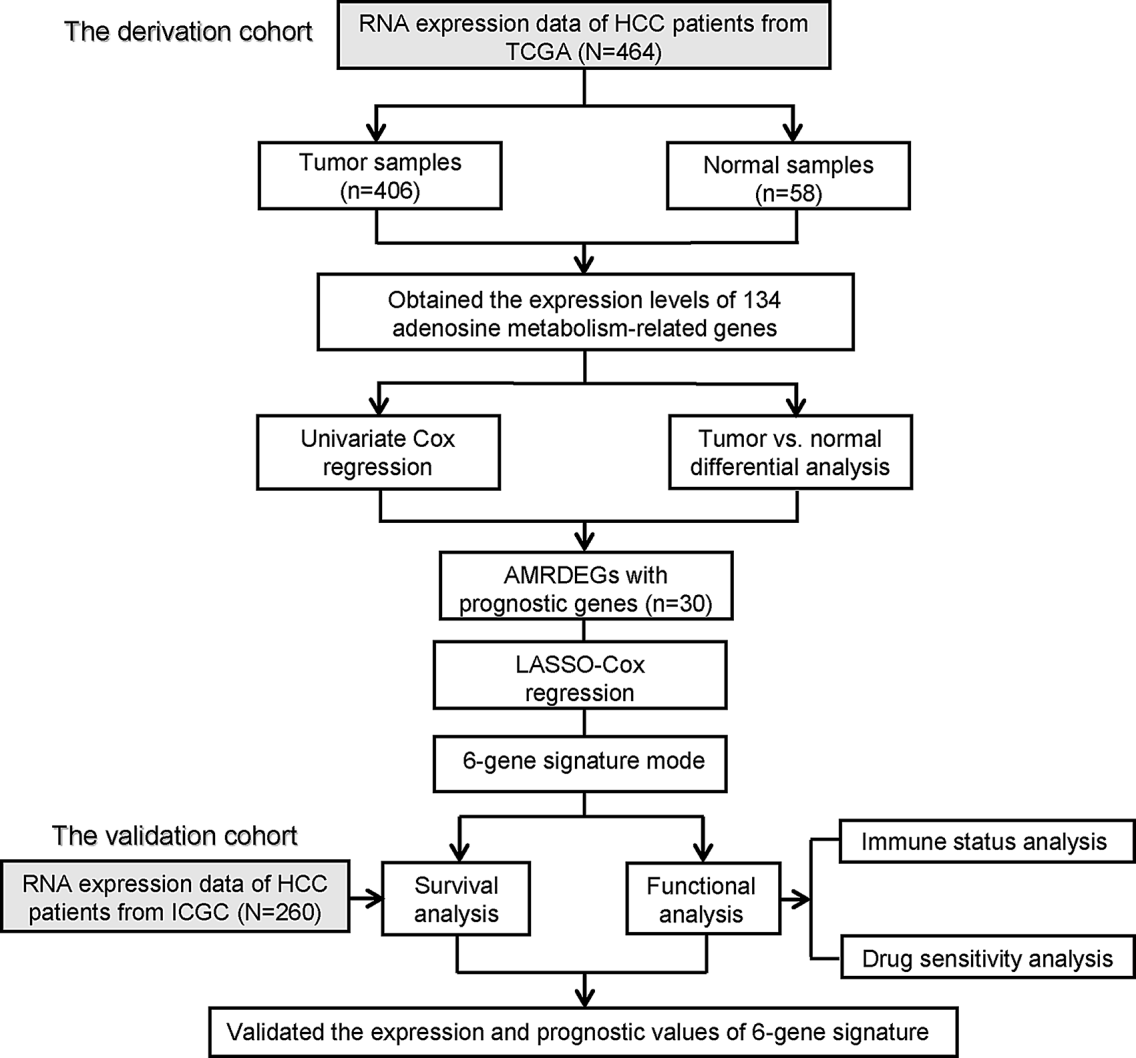
Flow chart of data collection and analysis

A visual representation of the study can be observed in Fig. 1, illustrating the inclusion of 464 samples from TCGA-LIHC and 260 samples from the ICGC (LIRI-JP) cohorts. Further information regarding the clinical characteristics of these patients can be found in Online Resource 2.

### Identification and construction of prognostic gene markers using the TCGA cohort

**Fig. 2:**
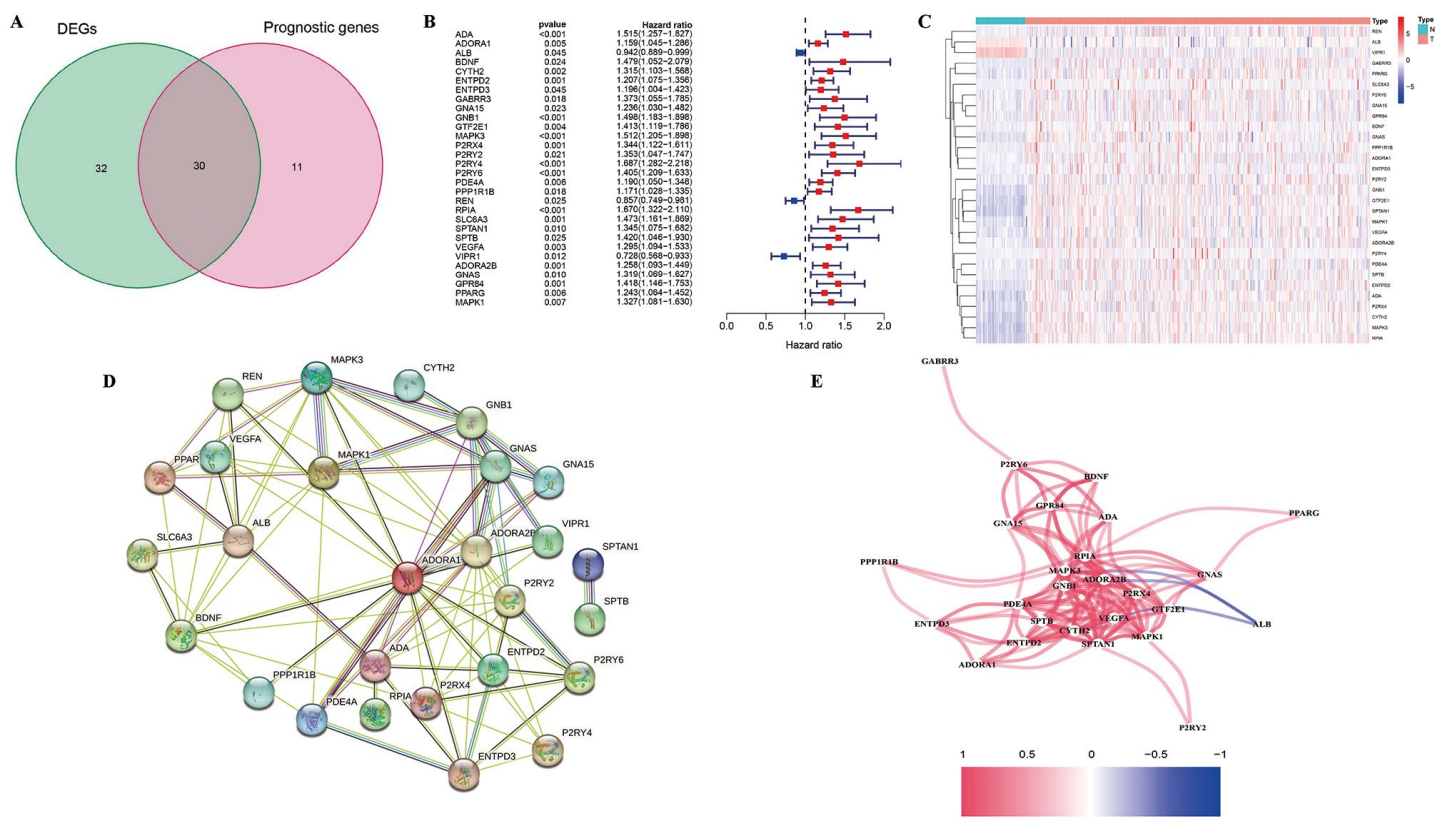
Adenosine metabolism-related differentially expressed genes (AMRDEGs) identified in HCC (**a**) Venn diagram illustrating the intersection of prognostic genes and differentially expressed genes (**b**) Forest plot of the relationship between the expression of AMRDEGs and overall survival in HCC based on univariate Cox regression analysis (**c**) Heatmap of the expression levels of AMRDEGs in HCC and normal liver tissue (**d**) Protein–protein interaction network of AMRDEGs (**e**) Gene expression correlation network of AMRDEGs

To assess the role of adenosine metabolism-related genes in HCC, 134 gene sets associated with adenosine metabolism were obtained from the existing literature (gene names and descriptions are provided in Online Resource 3). Using the TCGA database, we identified 62 DEGs related to adenosine metabolism in HCC and adjacent normal tissues. Based on the expression data of these gene sets, 41 prognostic adenosine metabolism-related genes were identified by univariate Cox analysis (Online Resource 4a). PCA analysis revealed significant differences in RNA sequencing data between tumors and normal tissues (Online Resource 4b), and the alterations in the expression of 41 genes in tumor tissues was depicted in Online Resource 4c. Further intersection of these genes with previously identified DEGs resulted in a final set of 30 AMRDEGs (Fig. 2a). The HRs of these 30 genes in relation to patient OS are shown in Fig. 2b. Among the identified AMRDEGs, three genes (ALB, REN, and VIPR1) with an HR < 1 were protective, whereas the remaining 27 genes with an HR > 1 were considered risk-associated. Expression patterns in normal and tumor tissues are shown in Fig. 2c. Protein interaction network analysis and gene expression correlation networks were used to analyze the interactions among these 30 AMRDEGs, which demonstrated significant positive correlations (Fig. 2d and e).

**Fig. 3:**
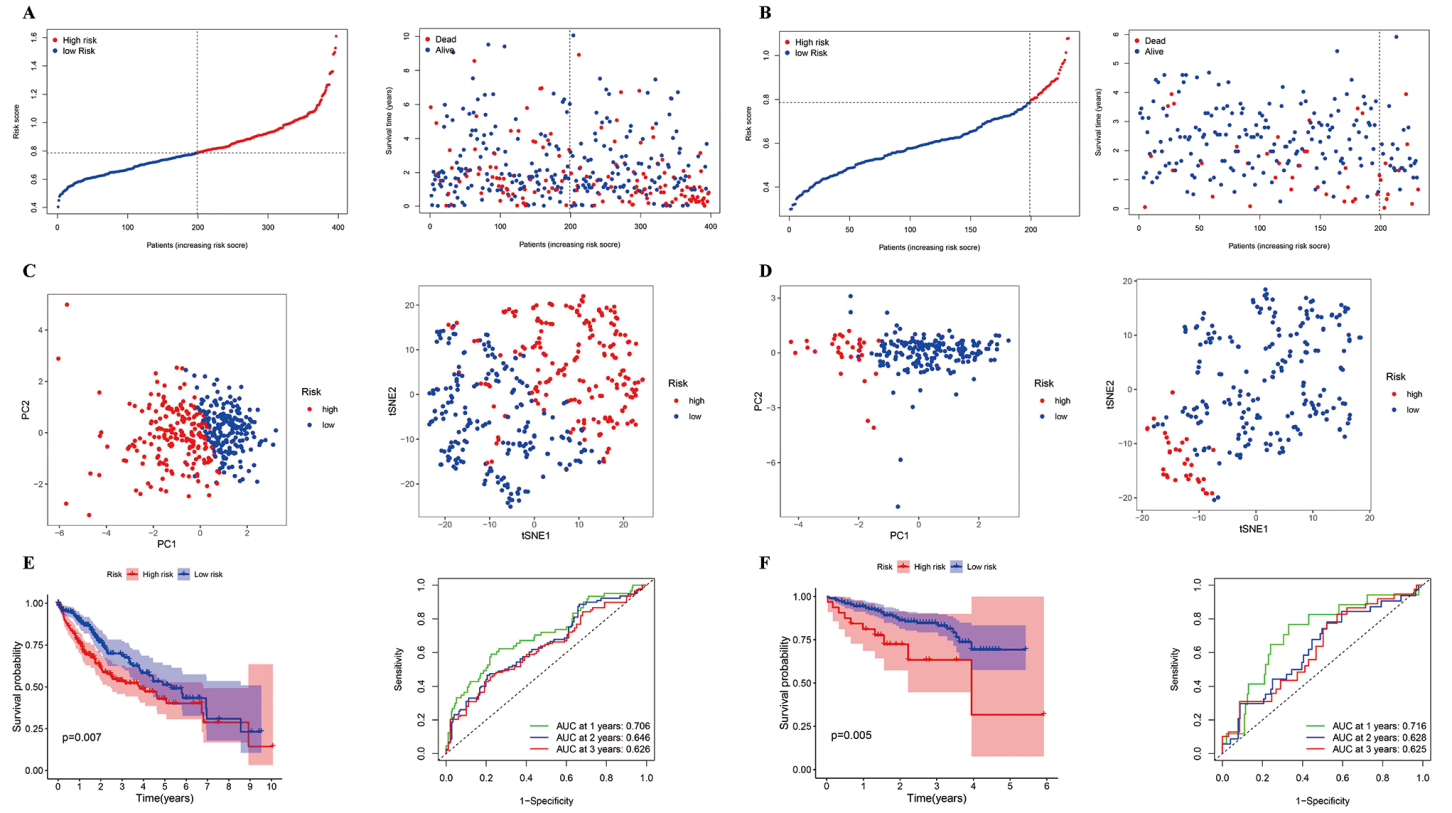
Construction and validation of the risk score made up of six genes in TCGA (**a**, **c**, and **e**) and ICGC (**b**, **d**, and **f**) datasets (**a**, **b**) Based on the median risk scores and individual gene expressions of the six genes, the samples were divided into high-risk and low-risk groups. Red for high risk; blue for low risk (left). Relationship between different HCC risk groups and survival time (right) (**c** and **d**) PCA (left) and t-SNE (right) analyses shows the sample distribution after dimensionality reduction according to expression levels (**e** and **f**) Kaplan–Meier analysis of the correlation between expression in high- and low-risk groups and OS in patients with HCC Red: high risk; blue: low risk (left). AUC curves of the risk score predicting 1-, 2-, and 3-year changes over time (right). Red: high risk, blue: low risk

Penalized LASSO-Cox PH regression (glmnet R Package) was used to analyze the expression level and prognosis of AMRDEGs in the training set (TCGA) to explore the linear relationship between genes. All combinations of lambda values are listed in Online Resource 5a and b, with lambda.min being 6, and the gene description and risk coefficient are shown in Online Resource 6. Based on the median risk scores for each sample in the training set, the samples were classified into high- and low-risk groups. Scatter plots generated using these risk scores to compare the differences between the high- and low-risk groups indicated that patients with a higher risk score may experience a shorter OS than those with a lower risk score (Fig. 3a). Additionally, principal component analysis (PCA) and t-distributed stochastic neighbor embedding (t-SNE) were employed for dimensionality reduction analysis on both datasets, resulting in distribution diagrams, where the red and blue areas represent high-risk and low-risk groups, respectively. Notably, statistically significant differences were observed between the groups (Fig. 3c). The relationship between individual gene expression levels and patient OS was analyzed to evaluate the impact of these six genes on HCC prognosis (Online Resource 7). As illustrated in Fig. 3, except for P2RY4 (*P* = 0.05), elevated expression levels of the remaining five genes, namely SLC6A3, VEGFA, ADA, P2RY6, and RPIA were significantly associated with shorter OS (< 0.001). Subsequently, Kaplan–Meier survival and time-dependent ROC curves were used to assess the predictive value of the patient prognosis risk score. As shown in Fig. 3e, the low-risk group exhibited a longer OS than the high-risk group. Moreover, the risk score displayed good predictive ability at 1, 2, and 3 years (AUC values of 0.706, 0.646, and 0.626, respectively).

### Validation of AMrisk in the ICGC database

To validate the prognostic potential of AMrisk in predicting HCC patient outcomes, a similar statistical analysis was conducted using an independent dataset from the ICGC. The AMrisk for each sample within the ICGC cohort was calculated and categorized into high-risk and low-risk groups based on the median values obtained from TCGA database. Scatter plots were then generated to visualize the groups (Fig. 3b). Notably, patients with higher risk scores exhibited a shorter OS than those in the low-risk group. Subsequent PCA and t-SNE consistently demonstrated a clear separation between samples belonging to the high- and low-risk groups within the ICGC cohort (Fig. 3d). Furthermore, Kaplan–Meier survival analysis confirmed that patients classified as high-risk displayed a trend towards reduced OS, similar to that observed in TCGA cohort. Additionally, ROC analysis revealed AUC values of 0.716, 0.628, and 0.625 at 1, 2, and 3 years, respectively (Fig. 3f).

### Prognostic value of AMrisk in HCC

**Fig. 4:**
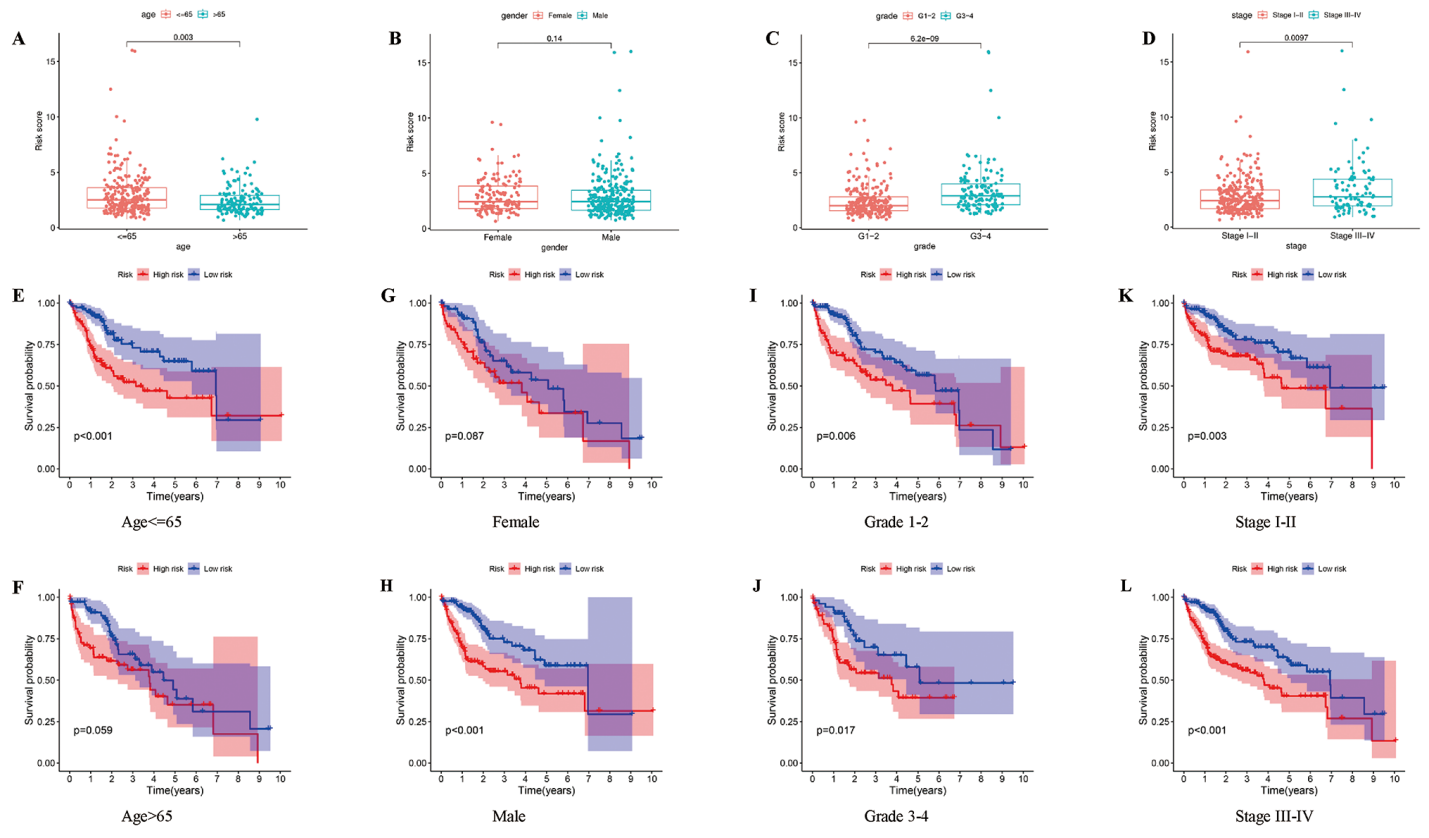
Inter-group comparison and Kaplan–Meier analysis were conducted to assess the risk scores in ATCG with diverse clinical features (**a**-**d**) Inter-group comparison of risk scores in stratification of clinical features, including age (**a**. red: age ≤ 65, blue: age > 65), sex (**b**. red: female, blue: male), grade (**c**. red: G1-2, blue: G3-4), and stages (**d**. red: stage I-II, blue: stage III-IV) (**e**-**l**) Kaplan-Meier analysis of clinical features in high- and low-risk groups (Red: high risk, blue: low risk) (**e** and **f**): Age ≤ 65 and age > 65; (**g** and **h**): female and male; (**i** and **j**): grade 1–2 and grade 3–4; (**k**-**j**): stage I-II and stage III-IV

To assess the predictive capability of AMrisk for prognosis in HCC across various clinical characteristics, patients were stratified based on age (≤ 65 or > 65), sex (female or male), tumor grade (1–2 or 3–4), and tumor stage (I-II or III-IV). Comparison of the risk scores elucidated that patients aged ≤ 65 with grade 3–4 tumors and stage III-IV had higher risk scores. However, no significant difference in the risk scores was observed between the sexes (Fig. 4a-d). Subsequently, Kaplan–Meier survival analysis performed within each subgroup demonstrated that OS rates were lower in high-risk score groups than in low-risk score groups among men patients aged ≤ 65 with tumor grades 1–2/3–4, and tumor stages I-II/III-IV (Fig. 4e-l). These findings demonstrate the robustness of AMrisk in predicting the prognosis of HCC patients with diverse clinical characteristics.

**Fig. 5:**
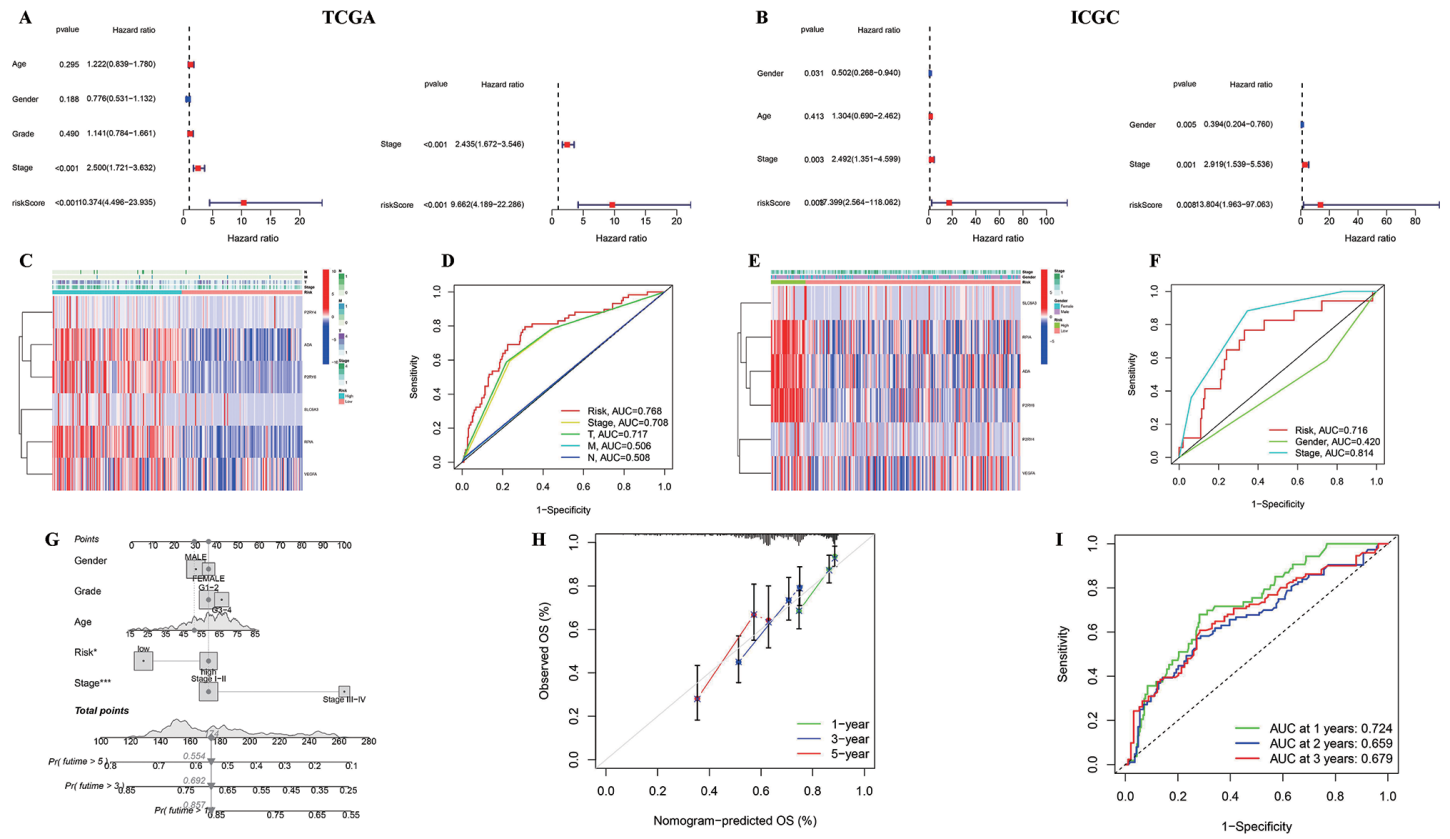
Construction and verification of a nomogram for the prognosis in HCC patients based on AMRisk (**a**) TCGA, (**b**) ICGC. Forest plots of univariate and multiple Cox regression analyses (**c**) TCGA, (**e**) ICGC Heatmap of the six adenosine metabolism-related differentially expressed genes (AMRDEGs) expression profiles and clinical characteristics of patients in high- and low-AMRisk groups (**d**) TCGA (**f**) ICGC Multi-index receiver operating characteristic (ROC) curves of the AMRisk and clinical characteristics (**g**) Prognosis nomogram (**h**) Calibration curves of the nomogram on 1-, 3-, and 5-year survival probability (**i**) ROC curves for 1-, 3-, and 5-year overall survival prediction of the nomogram

To determine the factors influencing survival in HCC patients, univariate and multivariate Cox regression analyses on AMrisk and the clinical characteristics that may be associated with OS were performed to assess whether AMrisk is an independent prognostic factor. A univariate Cox regression analysis on age, sex, grade (TCGA), and stage was initially performed, and OS-related prognostic features were identified for inclusion in the multivariate regression analysis. The results revealed that stage (HR = 2.435, 95% CI = 1.672–3.546, *P* < 0.001) in the TCGA cohort and sex (HR = 0.394, 95% CI = 0.204–0.760, *P* = 0.005) and stage (HR = 2.919, 95% CI = 1.539–5.536, *P* = 0.001) in the ICGC cohort were independent predictors of OS within their respective cohorts. Moreover, AMrisk emerged as an independent predictor in both cohorts: TCGA cohort, HR = 9.662, 95% CI = 4.189–22.286, *P* < 0.001. ICGC cohort, HR = 13.804, 95% CI = 1.963–97.063, *P* = 0.008). Notably, the HRs associated with AMrisk exceeded those associated with the other clinical characteristics (Fig. 5a and b). The heatmap of gene expression and clinical characteristics clearly demonstrated that all six AMRDEGs in the risk score exhibited significantly higher expression levels in the high-risk group, which was consistent with the trends observed in TCGA and ICGC databases (Fig. 5c and e). Furthermore, ROC curve analysis demonstrated that the AUCs for TCGA and ICGC were 0.768 and 0.716, respectively (Fig. 5d and f). These findings demonstrate that AMrisk is an independent prognostic risk factor for HCC with enhanced predictive ability.

### Construction and verification of a nomogram for prognosis based on AMrisk

To further improve the accuracy and predictive ability of the prediction model, a prognostic nomogram model was constructed by incorporating clinical characteristic data from TCGA database into AMrisk (Fig. 5g). The calibration curves were utilized to validate the accuracy of the prediction model, demonstrating a strong consistency between the predicted probabilities of survival rates at 1, 3, and 5 years and the actual observed survival outcomes (Fig. 5h). Additionally, calibration results and discrimination ability demonstrated by the AUC values of the nomogram were satisfactory (0.724, 0.659, and 0.679 for 1-, 2-, and 3-years, respectively; Fig. 5i).

### Biological functional enrichment analysis of amrdegs

**Fig. 6:**
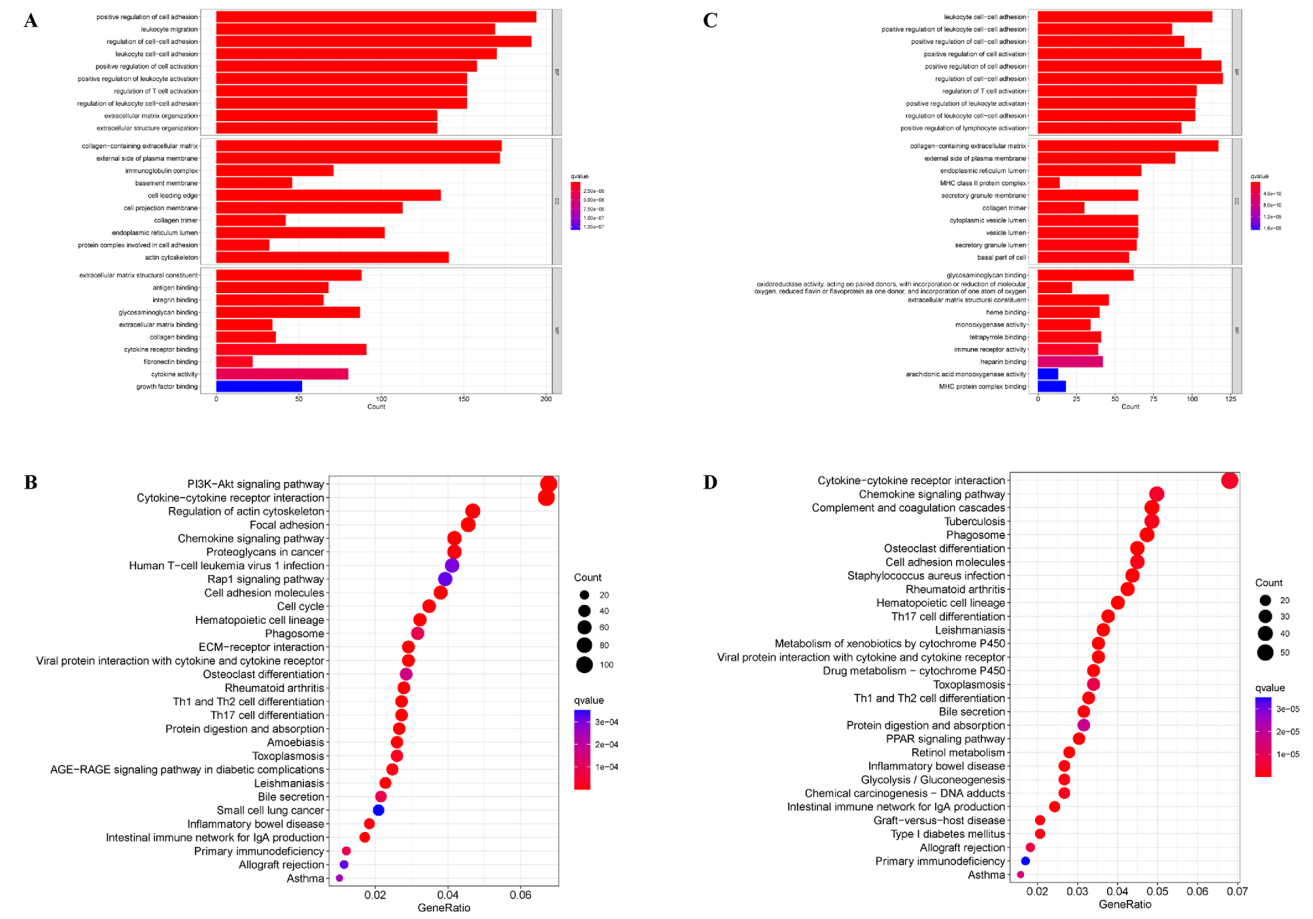
Biological Functional Enrichment Analysis of adenosine metabolism-related differentially expressed genes. **A** (TCGA), **C** (ICGC). Gene ontology (GO) enrichment analysis of adenosine metabolism-related differentially expressed genes (AMRDEGs). “BP” stands for “biological process”, “CC” stands for “cellular component” and “MF” stands for “molecular function”. **B** (TCGA), **D** (ICGC). Kyoto Encyclopedia of Genes and Genomes (KEGG) enrichment analysis of AMRDEGs. The color represents the statistical significance of the term. The size indicates the counts of enriched genes

GO and KEGG enrichment analyses of DEGs between the high- and low-risk groups in TCGA and ICGC cohorts were performed to investigate the biological functions associated with AMrisk. In the TCGA cohort, GO enrichment analysis revealed that DEGs were significantly enriched in several cell adhesion-related pathways, including positive regulation of cell adhesion, regulation of cell-cell adhesion, leukocyte cell-cell adhesion, and regulation of leukocyte cell-cell adhesion (Fig. 6a). Furthermore, KEGG enrichment analysis demonstrated that AMRDEGs were predominantly involved in crucial signaling pathways, such as the PI3K-Akt signaling pathway, cytokine-cytokine receptor interaction, regulation of the actin cytoskeleton, and focal adhesion (Fig. 6b). The results obtained for the ICGC cohort were consistent with those of the TCGA cohort, and several prominent pathways identified by GO enrichment analysis were associated with cell adhesion (Fig. 6c). Additionally, the pathways identified through the KEGG enrichment analysis were predominantly clustered into cytokine-cytokine receptor interactions, chemokine signaling pathways, complement and coagulation cascades, tuberculosis, and other related pathways (Fig. 6d). These findings suggest that AMRDEGs primarily participate in cellular adhesion and cytokine-cytokine receptor interactions.

### Immune status and tumor immune microenvironment in different risk groups

**Fig. 7:**
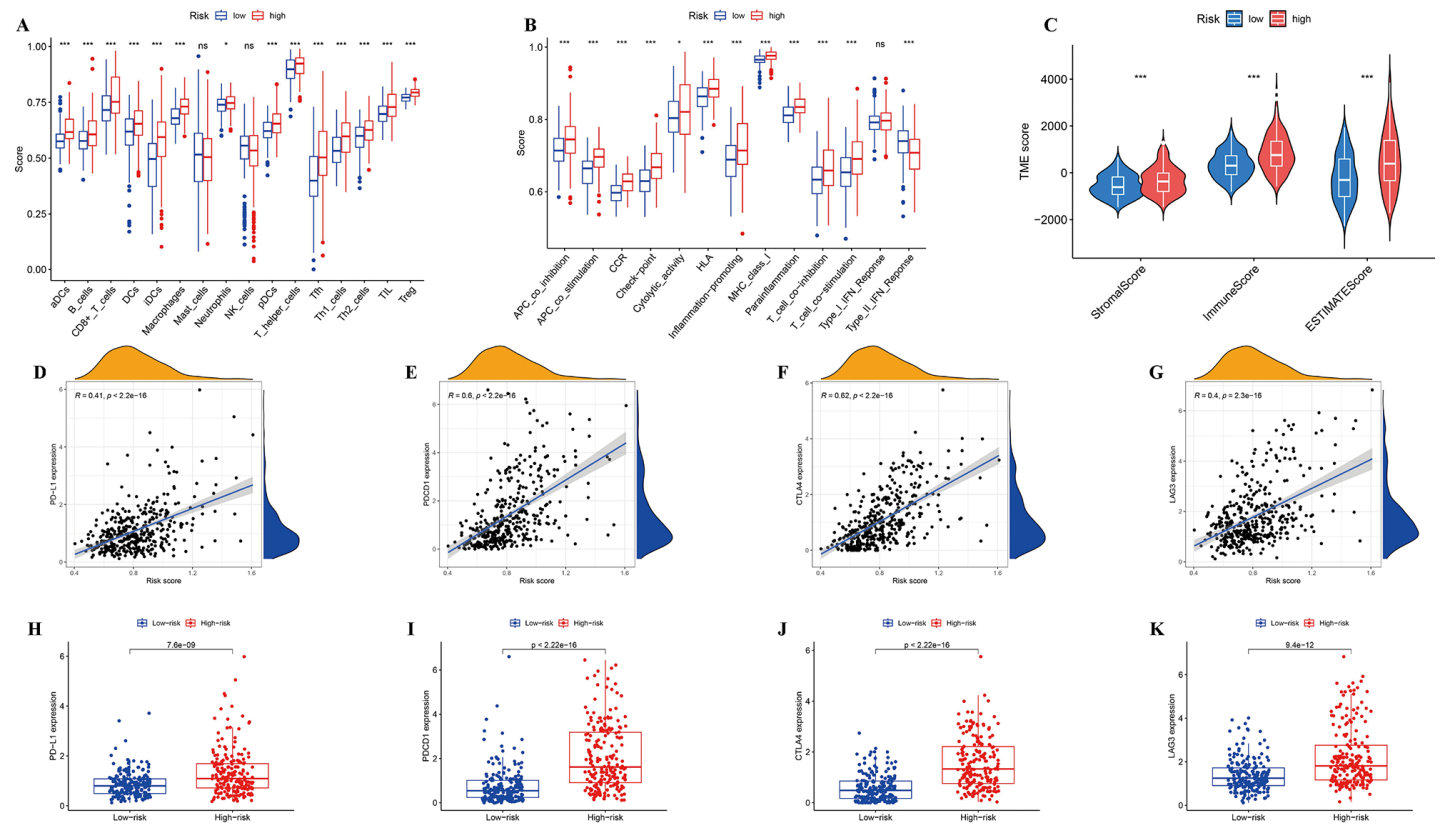
Immune status of high- and low-risk groups and association between tumor immune microenvironment and risk score in TCGA datasets **a**, **b** Single-sample gene set enrichment analysis (ssGSEA) (**a**, immune cell subpopulations; **b**, immune-related pathways) **c** ESTIMATE analysis **d**-**g** Correlation analysis between the risk score and the expression of immune checkpoint molecules (PD-1, PD-L1, CTLA4, and LAG3) **h**-**k** Comparison of the expression levels of the four immune checkpoint molecules in the high- and low-risk groups (red: high risk, blue: low risk, “*”*P* < 0.05, “**”*P* < 0.01, “***”P < 0.001, “ns” not significant)

To quantify the immune cell populations and immune functions associated with each sample’s gene set within the TCGA cohort, ssGSEA analysis was performed. Correlation analysis was then conducted between the ssGSEA scores of the high- and low-risk groups to investigate the potential associations between risk scores and immune responses. Compared to the low-risk group, a significant increase in innate immune cells and pathways related to immune function in the high-risk group was observed, including aDCs, B cells, and CD8 + T cells. Conversely, expression levels of the type II IFN response pathway, which is involved in immunoregulatory functions, were significantly lower in the high-risk group (Fig. 7a and b), indicating an activated state of immunity in the high-risk group.

Furthermore, both the high- and low-risk groups in this study were analyzed using the pan-cancer immune infiltration analysis data for HCC obtained from the official website of ESTIMATE (https://bioinformatics.mdanderson.org/estimate/index.html). Subsequently, the matrix, immune, and ESTIMATE scores for each group were calculated and compared. (Fig. 7c). Notably, the high-risk group exhibited significantly higher matrix, immune, and ESTIMATE scores. These results indicate a greater proportion of matrix cells and an enhanced level of immune infiltration in the high-risk group than in their low-risk counterparts.

PD-1/PD-L1 interaction is crucial for tumor progression, proliferation, and infiltration [30]. The expression levels of immune checkpoint molecules were compared between the high- and low-risk groups using data obtained from TCGA cohorts (Fig. 7d-k). The findings revealed significantly higher levels of PD-1, PD-L1, CTLA4, and LAG3 expression in the high-risk group than in the low-risk group, which positively correlated with AMrisk. Collectively, these results suggest a potential association between AMrisk and immune cell infiltration, immune-related functions, and the tumor immune microenvironment status in HCC.

### Validating the expression levels of genes involved in constructing the prognostic model

**Fig. 8:**
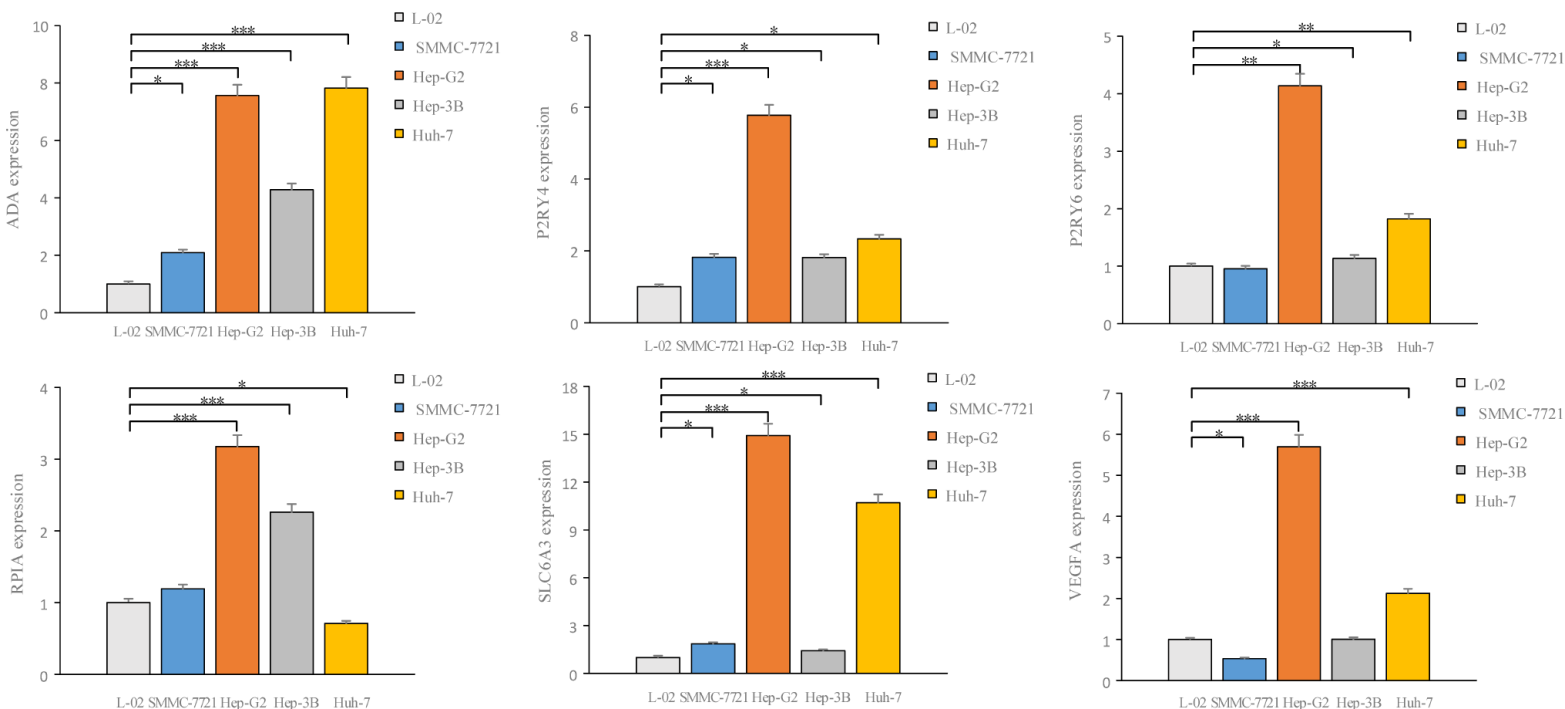
Verify the expression levels of 6 genes involved in constructing the prognostic model. L-02 is a normal human hepatocyte line, SMMC-7721, Hep-G2, Hep-3B and Huh-7 are human hepatocellular carcinoma cell. “*” stands for *P* < 0.05, “**” stands for *P* < 0.01, “***” stands for *P* < 0.001

To validate the expression changes in AMRDEGs involved in constructing the prognostic model, RT-PCR was performed to quantify the expression levels of these six genes in the normal liver cell line L-02 and the liver cancer cell lines SMMC-7721, Hep-G2, Hep-3B, and Huh-7. The results are shown in Fig. 8, which revealed that ADA, P2RY4, P2RY6, RPIA, and SLC6A3 genes exhibited significantly higher expression levels in Hep-G2, Hep-3B, and Huh-7 cells than in normal liver cells L-02; particularly pronounced elevation was observed in Hep-G2 cells. Compared to L-02 cells, ADA, P2RY4, and SLC6A3 were upregulated in SMMC-7721, whereas no significant differences were observed in P2RY6 and RPIA. Although VEGFA expression was decreased in SMMC-7721, it was elevated in Hep-G2 and Huh-7 cell lines. The enhanced expression trend of these six AMRDEGs observed in tumor cells aligns with the previously proposed predictions made in this study and further verifies the predictive ability of the prognostic model.

### Prediction of drug sensitivity to commonly targeted drugs in different risk groups for HCC patients

**Fig. 9:**
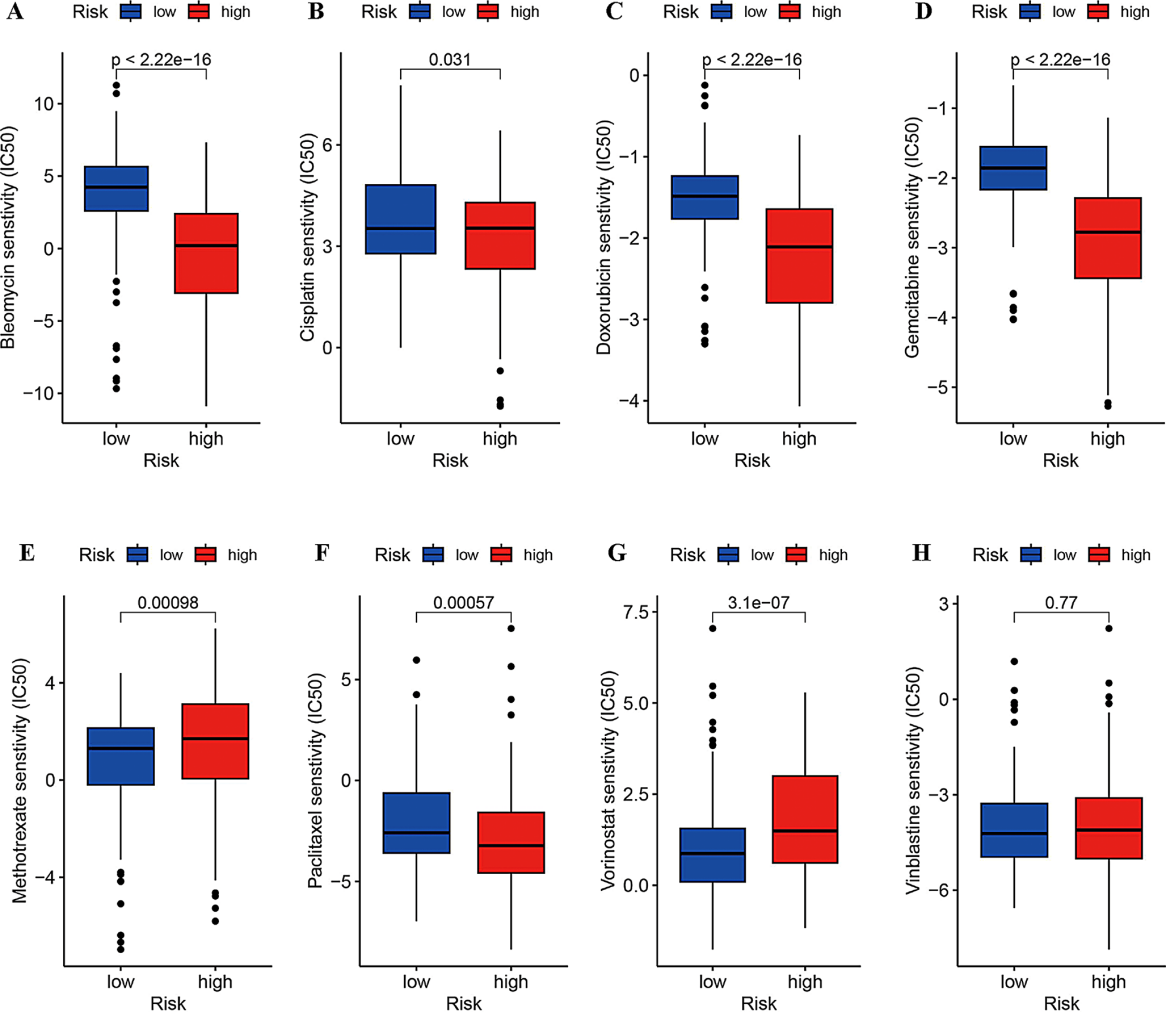
Prediction of drug sensitivity to 8 targeted drugs in different risk groups for HCC patients. Bleomycin, cisplatin, doxorubicin, gemcitabine, methotrexate, paclitaxel, vorinostat, and vinblastine, respectively. Red: high risk, blue: low risk

Because chemotherapy and molecular targeted drugs have been employed as first-line therapies for systemic HCC patients, the sensitivity of high- and low-risk patients to eight frequently used antineoplastic drugs was predicted to provide clinical guidance for drug selection in distinct risk groups. The results demonstrated that high-risk group exhibited higher sensitivity to methotrexate and vorinostat and lower sensitivity to bleomycin, cisplatin, doxorubicin, gemcitabine, and paclitaxel. However, no significant difference in the sensitivity to vinblastine was observed in the high-risk group (Fig. 9).

## Discussion

Although numerous predictive models for HCC have been developed, such as the HCC prognostic model established by Shi et al. [31], which is based on four genes associated with exhausted CD8 + T cells and has demonstrated its potential in HCC prognosis and guidance of immunotherapy, and the prognostic model proposed by Chen et al. [32], who conducted gene and molecular studies on the anoikis-related pathway and identified five genes as prognostic markers for HCC, capable of predicting OS to a certain extent. Study by Yuqiao Chen’s [33] group further discovered a novel cuproptosis-related gene signature that can predict the prognosis of HCC patients. By performing bioinformatics analysis using transcriptome data and clinical information from 373 HCC patients in the TCGA database, they established a prediction model indicating that deficiency of PDXK gene may lead to copper apoptosis in HCC. Nevertheless, the stringent prerequisites for the development of novel therapeutic targets involving key genes necessitate extensive foundational research to explore more comprehensive metabolic mechanisms. Consequently, we opted to explore a nascent field at the outset of this study. Herein, we have successfully established and validated a novel prognostic model based on adenosine metabolism genes, thereby offering a fresh avenue for HCC treatment and laying down an initial theoretical foundation for future investigations. Studies on reliable biomarkers based on the biological characteristics of the adenosine metabolism pathway are scarce. This study systematically investigated altered expression patterns within the adenosine signaling pathway in HCC and explored their potential association with patient prognosis.

The production of immunosuppressive adenosines in the TME is crucial for immune escape [34]. Extracellular adenosine substantially affects the distribution and function of immune cells within the TME by binding to adenosine receptors on these cells [8, 35]. However, the exact mechanism and the abnormal expression patterns of key genes involved in adenosine metabolism, namely adenosine kinase (ADK) and equilibrative nucleoside transporter type 4 (ENT4), in clinical liver cancer cases have been recently reported [8]. Downregulation of ADK expression in human HCC may further enhance intracellular adenosine accumulation, whereas upregulation of ENT4 expression facilitates extracellular export along concentration gradients. Therefore, extracellular adenosine inhibited the proliferation and promoted the death of CD8 + and CD4 + T cells in a dose-dependent manner. Furthermore, adenosine potentially hampers anti-tumor immunity by upregulating Treg genes and genes associated with the negative regulation of T cell proliferation. However, whether the level of adenosine within the TME correlates with tumor proliferation and patient prognosis remains unexplored.

In this study, we investigated alterations in adenosine metabolism-related genes and their correlation with clinicopathological features in HCC by analyzing RNA sequencing data and the clinical characteristics of HCC patients. LASSO-Cox regression analysis was used to construct AMrisk, which comprised six AMRDEGs: ADA, P2RY4, P2RY6, RPIA, SLC6A3, and VEGFA. Based on the findings shown in Fig. 2b and Online Resource 7, all six genes were associated with an increased risk of HCC. Furthermore, the expression levels of five of these six genes were strongly correlated with a poor prognosis (*P* < 0.001), with the exception of P2RY4 (*P* = 0.05). ADA is responsible for encoding adenosine deaminase, an enzyme that facilitates the hydrolytic deamination of adenosine and 2-deoxyadenosine. It plays a crucial role in regulating purine metabolism and maintaining adenosine homeostasis [36–39]. ADA increases the immunogenicity of dendritic cells by modulating the expression of costimulatory molecules and secretion of cytokines and chemokines, while also promoting differentiation and proliferation of CD4 + T-cells [40]. Different mutations have been reported in this particular gene and are associated with human disorders characterized by compromised immune function, including severe combined immunodeficiency disease (SCID), a condition caused by a lack of adenosine deaminase [41]. A significant increase in adenosine deaminase levels has been demonstrated in various tumor tissues, including cholangiocarcinoma, lymphoma, diffuse large B-cell lymphoma, and pancreatic adenocarcinoma. In most cancer types, adenosine deaminase is positively correlated with infiltrating immune cells, and its elevated expression may serve as an indicator of poor survival rates [42, 43]. The protein encoded by P2RY4 is classified as a G-protein-coupled receptor that activates the phosphatidylinositol-calcium second messenger system through G protein coupling, playing a crucial role in chloride ion transport within the jejunum epithelium [44]. Similar to P2RY4, P2RY6 belongs to the G-protein-coupled receptor family and exerts similar functions. P2RY6 is significantly upregulated in various cancer tissues, including HCC, potentially influencing immune-related pathways such as cytokine-cytokine receptor interactions [45]. Furthermore, high P2RY6 expression is closely associated with unfavorable prognoses [46, 47]. RPIA encodes ribose 5-phosphate isomerase, an enzyme that catalyzes the reversible conversion of ribose-5-phosphate to ribulose 5-phosphate and plays a crucial role in the initial step of the non-oxidative branch of the pentose phosphate pathway. Despite the limited research on the involvement of RPIA in tumorigenesis, recent findings in pancreatic ductal adenocarcinoma have demonstrated its regulatory function in directing glucose intermediates towards the DNA/RNA biosynthesis pathway and influencing tumor growth, suggesting its potential as a pivotal player in cancer biology [48]. The expression of the SLC6A3 gene impacts dopamine and catecholamine metabolism and plays a crucial role in regulating dopamine transporters; however, its role in tumor occurrence and development remains unclear. Increased expression of this gene is associated with poor prognosis in various cancers [49, 50]). VEGFA is a member of the vascular endothelial growth factor family and produces a protein that binds to heparin. This protein stimulates the proliferation and movement of vascular endothelial cells, which is crucial for both normal and abnormal blood vessel formation. VEGF-A has been overexpressed in many human tumors, including liver cancer, and increased expression of this gene is associated with tumor progression, recurrence, and survival rates [51, 52]. The undeniable role of VEGFA in tumor development is well-established. Bevacizumab, a current drug targeting VEGFA, has been approved as part of immunotherapeutic combinations for HCC and has demonstrated remarkable anti-tumor efficacy [53–55]. These findings strongly support the significance of VEGFA in HCC. In summary, these genes may serve as independent risk factors for HCC occurrence and development, while highlighting the significance of constructing an AMrisk model based on these genes to predict HCC patient prognosis.

To further investigate the biological functions and regulatory pathways associated with the changes in adenosine metabolism in HCC, functional enrichment analysis using GO was performed, which revealed that DEGs in the high- and low-risk groups were primarily enriched in several cell adhesion-related biological pathways and molecular functions related to extracellular matrix structural constituents. KEGG analysis identified the PI3K-Akt signaling pathway as the most crucial pathway. PI3K-Akt signaling pathway is involved in HCC cell proliferation and anti-apoptotic processes [56, 57]. Furthermore, the regulation of cell adhesion and cytoskeletal reorganization is crucial for tumor cell invasion and migration [58, 59]. Consistent with the GO analysis results, the KEGG analysis indicated the involvement of these DEGs in actin cytoskeleton regulation and the focal adhesion signaling pathway. Subsequently, ssGSEA was used for comparing the immune cell and immune function scores between the high- and low-risk groups. Our findings demonstrated a significant increase in various immune cells, including aDCs and B cells, and multiple immune function-related pathways, such as APC co-inhibition and co-stimulation, in the high-risk group, suggesting that increased immune infiltration may contribute to a poor prognosis in this population. Enhanced immune responses are typically associated with antitumor activity, but in certain cases, persistent and excessive immune responses may result in chronic inflammation or immune evasion, which can instead promote tumor progression and drug resistance. For instance, studies have demonstrated that tumor-associated macrophages within the tumor microenvironment can be reprogrammed by tumors to facilitate tumor cell growth, angiogenesis, and immune evasion [60]. Therefore, an augmented immune response may be accompanied by a higher risk of tumorigenesis depending on the specific immunological status of the tumor microenvironment [61]. Conversely, expression of the Type II IFN response pathway was significantly lower in the high-risk group, consistent with those of previous studies that reported defects in the IFN signaling pathway in liver cancer cells [62]. ESTIMATE analysis demonstrated a significantly higher immune cell infiltration and stromal cell ratio in HCC tissues of the high-risk group than in those of the low-risk group, providing further evidence of immune activation and enhanced immune-related pathways in the high-risk group.

Immunotherapy utilizing ICIs has emerged as a novel and practical therapeutic approach for HCC patients. Our findings revealed that the expression levels of PD-1, PD-L1, CTLA4, and LAG3 were significantly elevated in the high-risk group and were positively correlated with AMrisk, suggesting that the high-risk group may have a greater response to immunotherapy. In the subsequent targeted drug sensitivity prediction analysis, the high-risk group exhibited elevated sensitivity to methotrexate and vorinostat and reduced sensitivity to bleomycin, cisplatin, doxorubicin, gemcitabine, and paclitaxel. These findings may provide potential insights for tailoring personalized clinical treatment for HCC patients.

The study also possesses certain limitations, our primary focus was on elucidating the differential expression patterns of these genes in HCC and normal tissues. However, due to the inherent limitations of current technological advancements and methodologies, establishing a direct causal relationship between these observed differences and the involvement of stromal/immune cells remains an arduous task.

## Conclusion

In summary, our study findings elucidated a novel association between changes in adenosine metabolism-related regulatory genes and clinicopathological features, and prognosis in HCC. By screening six AMR-DEGs (ADA, P2RY4, P2RY6, RPIA, SLC6A3, and VEGFA), we developed and validated a prognostic model for HCC. Furthermore, the relationships between AMrisk and immune cell infiltration, immune-related functions, and immune checkpoint activation were analyzed to provide valuable insights into HCC immunotherapy strategies. Additionally, the expression levels of these six genes in normal liver cells and liver cancer cells were validated, while predicting their sensitivity to commonly used antitumor drugs. These findings could potentially contribute to the development of novel strategies for the clinical treatment of HCC.

## Electronic supplementary material

Below is the link to the electronic supplementary material.

Supplementary Material 1

## Data Availability

No datasets were generated or analysed during the current study.

## Abbreviations

HCC: Hepatocellular carcinoma
OS: Overall survival
DEGs: Differentially expressed genes
AMrisk: Adenosine metabolism-related risk score
LASSO: Least absolute shrinkage and selection operator
qRT-PCR: Quantitative reverse transcription polymerase chain reaction
TME: Tumor microenvironment
ICIs: Immune checkpoint inhibitors
PD-1: Programmed cell death receptor 1
PD-L1: Programmed cell death receptor ligand 1
CTLA-4: Cytotoxic T-lymphocyte-associated protein 4
LAD-3: Lymphocyte-activated gene-3
TCGA: The Cancer Genome Atlas
AMRDEGs: Adenosine metabolism-related differentially expressed genes
AMrisk: Adenosine metabolism-related risk score
ICGC: International Cancer Genome Consortium
LASSO: least absolute shrinkage and selection operator
AUC: Areas under the curve
ROC: Receiver operating characteristic
HRs: Hazard ratios
CIs: Confidence intervals
GO: Gene Ontology
KEGG: Kyoto Encyclopedia of Genes and Genomes
ESTIMATE: Estimation of Stromal and Immune cells in MAlignant Tumor tissues using Expression data
PCA: Principal component analysis
t-SNE: t-distributed stochastic neighbor embedding
