# Supplementary material for: Prognostic model development using novel genetic signature associated with adenosine metabolism and immune status for patients with hepatocellular carcinoma
Source: J Physiol Biochem. 2024 Nov 15;81(1):157–72. doi: 10.1007/s13105-024-01061-8 (PMC11958414; doi:10.1007/s13105-024-01061-8)
Supplement: Supplementary file 1 — Supplementary Material 1 [file 13105_2024_1061_MOESM1_ESM.docx]

**Supplementary materials**

**Online Resource 1. Primers for Real-Time PCR**

| Name | Sequences (5’-3’) |
| --- | --- |
| ADA-F | GCCTTCGACAAGCCCAAAGTA |
| ADA-R | CTCTGCTGTGTTAGCTGGGAG |
| P2RY4-F | GGAGCTGGACTGTTGGTTTGA |
| P2RY4-R | CATAGGGTTGGGGCGTTAAGG |
| P2RY6-F | GTGTCTACCGCGAGAACTTCA |
| P2RY6-R | CCAGAGCAAGGTTTAGGGTGTA |
| RPIA-F | AGTGCTGGGAATTGGAAGTGG |
| RPIA-R | GGGAATACAGACGAGGTTCAGA |
| SLC6A3-F | TTTCTCCTGTCCGTCATTGGC |
| SLC6A3-R | TGAAGCCCACACCTTTCAGTAT |
| VEGFA-F | AGGGCAGAATCATCACGAAGT |
| VEGFA-R | AGGGTCTCGATTGGATGGCA |

**Online Resource 2. Clinical characteristics of the HCC patients used in this study.**

| **Characteristics** | **TCGA cohort**  **(N=403)** | | **ICGC cohort**  **(N=260)** | |
| --- | --- | --- | --- | --- |
| **Fustat** |  | |  | |
| Alive | 257 (63.8%) | | 214 (82.3%) | |
| Dead | 146 (36.2%) | | 46 (17.7%) | |
| **Age at diagnosis(years)** | |  | |  |
| ≤65 | 232 (57.6%) | | 98 (37.7%) | |
| >65 | 138 (34.2%) | | 162 (62.3%) | |
| Unknow | 33 (8.2%) | | NA | |
| **Gender** |  | |  | |
| Female | 139 (34.5%) | | 68 (26.2%) | |
| Male | 264 (65.5%) | | 192 (73.8%) | |
| **Stage** |  | |  | |
| Stage I+II | 283 (70.2%) | | 157 (60.4%) | |
| Stage III+IV | 96 (23.8%) | | 103 (39.6%) | |
| Unknow | 24 (6.0%) | | NA | |
| **Grade** |  | |  | |
| Grade 1+2 | 232 (57.5%) | | NA | |
| Grade 3+4 | 134 (33.3%) | | NA | |
| Unknow | 37 (9.2%) | | NA | |

**Online Resource 3. Adenosine metabolism-related genes and descriptions**

| **Gene Symbol** | **Gene Description** |
| --- | --- |
| ADA | adenosine deaminase |
| ADA2 | Adenosine Deaminase 2 |
| ADCY1 | adenylate cyclase 1 |
| ADCY2 | adenylate cyclase 2 |
| ADCY3 | adenylate cyclase 3 |
| ADCY4 | adenylate cyclase 4 |
| ADCY5 | adenylate cyclase 5 |
| ADCY6 | adenylate cyclase 6 |
| ADCY7 | adenylate cyclase 7 |
| ADCY8 | adenylate cyclase 8 |
| ADCY9 | adenylate cyclase 9 |
| ADK | adenosine kinase |
| ADO | 2-aminoethanethiol dioxygenase |
| ADORA1 | adenosine A1 receptor |
| ADORA2A | adenosine A2a receptor |
| ADORA2B | adenosine A2b receptor |
| AGPS | alkylglycerone phosphate synthase |
| AKT1 | AKT serine/threonine kinase 1 |
| ALB | albumin |
| ALDH7A1 | aldehyde dehydrogenase 7 family member A1 |
| APP | amyloid beta precursor protein |
| ARRB1 | arrestin beta 1 |
| ATP12A | ATPase H+/K+ transporting non-gastric alpha2 subunit |
| BDNF | brain derived neurotrophic factor |
| CALCA | calcitonin related polypeptide alpha |
| CALM3 | calmodulin 3 |
| CASP1 | caspase 1 |
| CASP3 | caspase 3 |
| CCL3 | C-C motif chemokine ligand 3 |
| CCR7 | C-C motif chemokine receptor 7 |
| CD81 | CD81 molecule |
| CNR1 | cannabinoid receptor 1 |
| COL3A1 | collagen type Ill alpha 1 chain |
| COMT | catechol-O-methyltransferase |
| CREB1 | CAMP responsive element binding protein 1 |
| CST7 | cystatin F |
| CXCL8 | C-X-C motif chemokine ligand 8 |
| CXCR4 | C-X-C motif chemokine receptor 4 |
| CYBB | cytochrome b-245 beta chain |
| CYTH2 | cytohesin 2 |
| DRD2 | dopamine receptor D2 |
| EDN1 | endothelin 1 |
| ENTPD1 | ectonucleoside triphosphate diphosphohydrolase 1 |
| ENTPD2 | ectonucleoside triphosphate diphosphohydrolase 2 |
| ENTPD3 | ectonucleoside triphosphate diphosphohydrolase 3 |
| ENTPD8 | ectonucleoside triphosphate diphosphohydrolase 8 |
| FOS | Fos proto-oncogene, AP-1 transcription factor subunit |
| FOXP3 | forkhead box P3 |
| GABBR1 | gamma-aminobutyric acid type B receptor subunit 1 |
| GABRR3 | gamma-aminobutyric acid type A receptor subunit rho3 |
| GCG | glucagon |
| GDNF | glial cell derived neurotrophic factor |
| GFAP | glial fibrillary acidic protein |
| GNA11 | G protein subunit alpha 11 |
| GNA15 | G protein subunit alpha 15 |
| GNAS | GNAS complex locus |
| GNB1 | G protein subunit beta 1 |
| GNB3 | G protein subunit beta 3 |
| GNG2 | G protein subunit gamma 2 |
| GPI | glucose-6-phosphate isomerase |
| GPR37 | G protein-coupled receptor 37 |
| GPR84 | G protein-coupled receptor 84 |
| GPR88 | G protein-coupled receptor 88 |
| GRK2 | G Protein-Coupled Receptor Kinase 2 |
| GRM1 | glutamate metabotropic receptor 1 |
| GRM2 | glutamate metabotropic receptor 2 |
| GRM5 | glutamate metabotropic receptor 5 |
| GTF2E1 | general transcription factor IlE subunit 1 |
| HCRT | hypocretin neuropeptide precursor |
| IFITM5 | interferon induced transmembrane protein 5 |
| IL10 | interleukin 10 |
| IL6 | interleukin 6 |
| INS | insulin |
| KNG1 | kininogen 1 |
| LAG3 | lymphocyte activating 3 |
| LTBP1 | latent transforming growth factor beta binding protein 1 |
| LTBP2 | latent transforming growth factor beta binding protein 2 |
| LTBP3 | latent transforming growth factor beta binding protein 3 |
| MAOB | monoamine oxidase B |
| MAPK1 | mitogen-activated protein kinase 1 |
| MAPK3 | mitogen-activated protein kinase 3 |
| MPO | myeloperoxidase |
| NECAB2 | N-terminal EF-hand calcium binding protein 2 |
| NGF | nerve growth factor |
| NOS3 | nitric oxide synthase 3 |
| NPS | neuropeptide S |
| NPY | neuropeptide Y |
| NT5E | 5'-nucleotidase ecto |
| NTRK2 | neurotrophic receptor tyrosine kinase 2 |
| NTS | neurotensin |
| P2RX1 | purinergic receptor P2X 1 |
| P2RX2 | purinergic receptor P2X 2 |
| P2RX3 | purinergic receptor P2X 3 |
| P2RX4 | purinergic receptor P2X 4 |
| P2RX5 | purinergic receptor P2X 5 |
| P2RX7 | purinergic receptor P2X 7 |
| P2RY1 | purinergic receptor P2Y1 |
| P2RY11 | purinergic receptor P2Y11 |
| P2RY12 | purinergic receptor P2Y12 |
| P2RY13 | purinergic receptor P2Y13 |
| P2RY14 | purinergic receptor P2Y14 |
| P2RY2 | purinergic receptor P2Y2 |
| P2RY4 | pyrimidinergic receptor P2Y4 |
| P2RY6 | pyrimidinergic receptor P2Y6 |
| PANX1 | pannexin 1 |
| PDE4A | phosphodiesterase 4A |
| PDYN | prodynorphin |
| PNP | purine nucleoside phosphorylase |
| PPARG | peroxisome proliferator activated receptor gamma |
| PPP1R1B | protein phosphatase 1 regulatory inhibitor subunit 1B |
| PTGER4 | prostaglandin E receptor 4 |
| PTGS2 | prostaglandin-endoperoxide synthase 2 |
| RAPGEF3 | Rap guanine nucleotide exchange factor 3 |
| RBFOX3 | RNA binding fox-1 homolog 3 |
| REN | renin |
| RPIA | ribose 5-phosphate isomerase A |
| SAG | S-antigen visual arrestin |
| SCT | secretin |
| SLC29A1 | solute carrier family 29 member 1(Augustine bloodgroup) |
| SLC29A2 | solute carrier family 29 member 2 |
| SLC6A3 | solute carrier family 6 member 3 |
| SP9 | Sp9 transcription factor |
| SPECC1L-ADORA2A | SPECC1L-ADORA2A readthrough(NMD candidate) |
| SPTAN1 | spectrin alpha, non-erythrocytic 1 |
| SPTB | spectrin beta, erythrocytic |
| TAC1 | tachykinin precursor 1 |
| TH | tyrosine hydroxylase |
| TNF | tumor necrosis factor |
| TRIM33 | tripartite motif containing 33 |
| USP4 | ubiquitin specific peptidase 4 |
| VEGFA | vascular endothelial growth factor A |
| VIP | vasoactive intestinal peptide |
| VIPR1 | vasoactive intestinal peptide receptor 1 |
| WDTC1 | WD and tetratricopeptide repeats 1 |


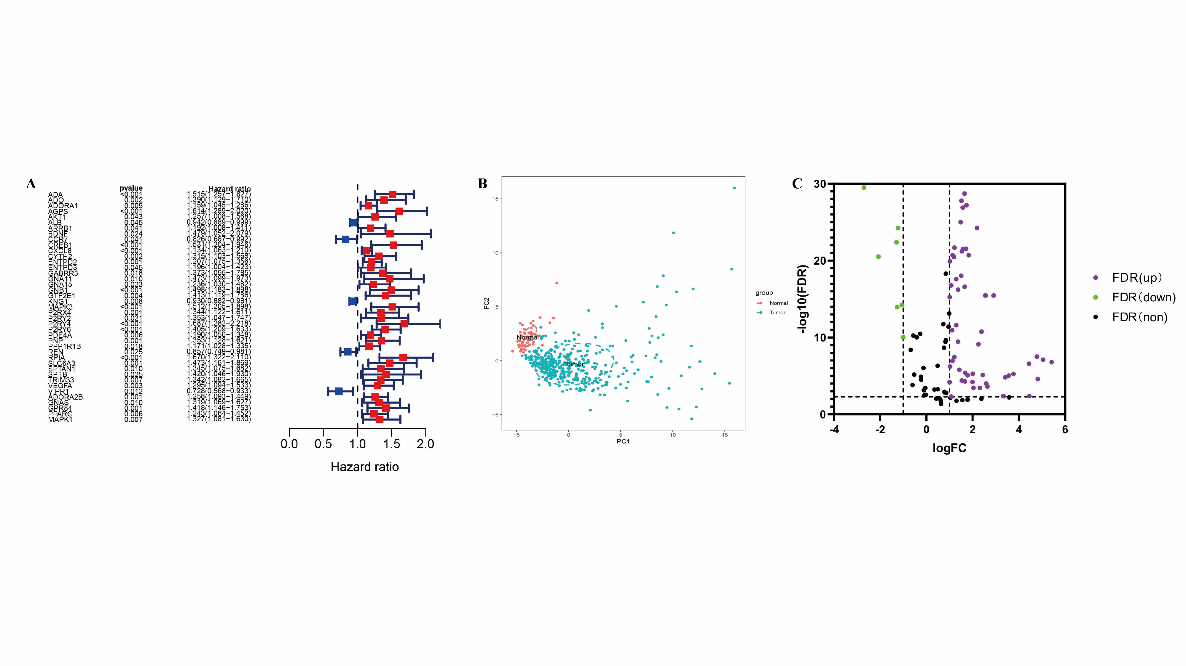
 **Online Resource 4.** 41 adenosine metabolism-related genes associated with prognosis A. Forest plot of gene expression and prognosis based on univariate Cox regression analysis. B. Principal component analysis (PCA). C. Volcano plot (adjusted p < 0.05 and |log2 (fold change) | > 1, purple/green represents up-/down-regulated genes).
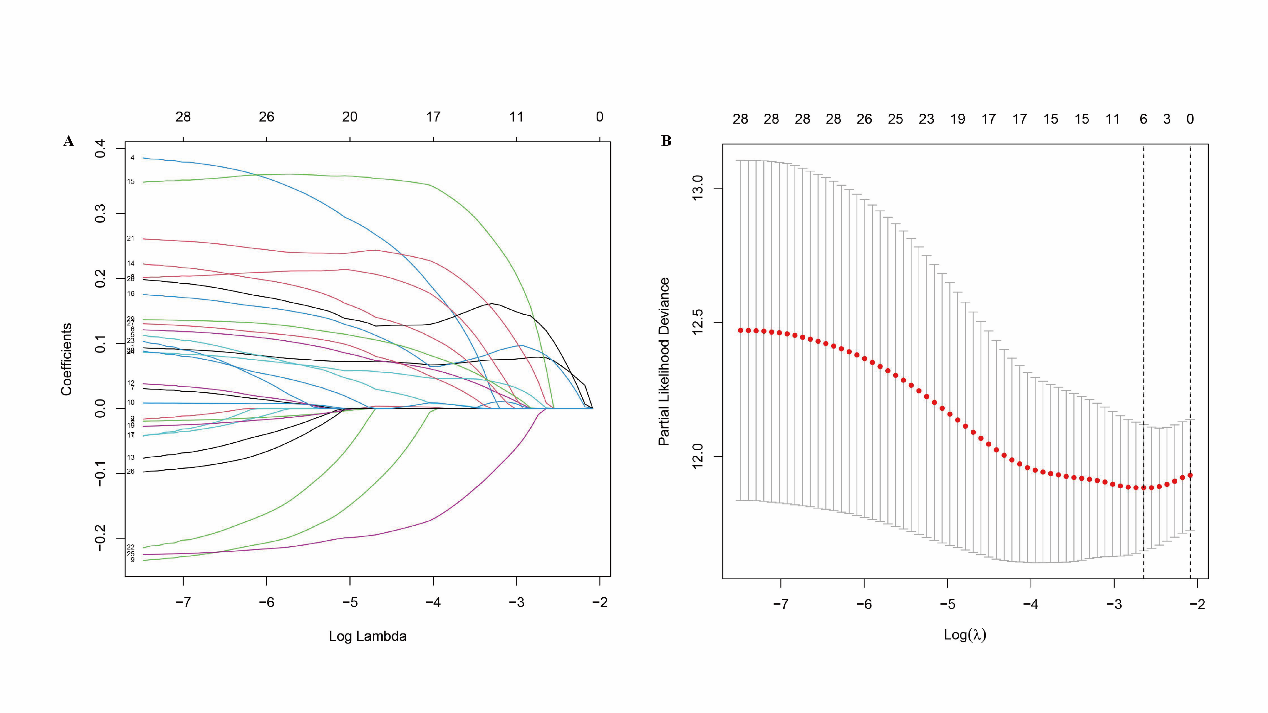
 **Online Resource 5.** The least absolute shrinkage and selection operator (LASSO)-Cox regression analysis. A. The coefficient spectrum of LASSO-Cox regression analysis. B. The tuning parameter (λ) in the LASSO model was selected by ten-fold cross-validation based on the minimum criterion. The minimum criterion and one standard error of the minimum criterion were used to draw a dotted perpendicular line at the optimal value.





**Online Resource 6.** The adenosine metabolism-related differentially expressed genes.
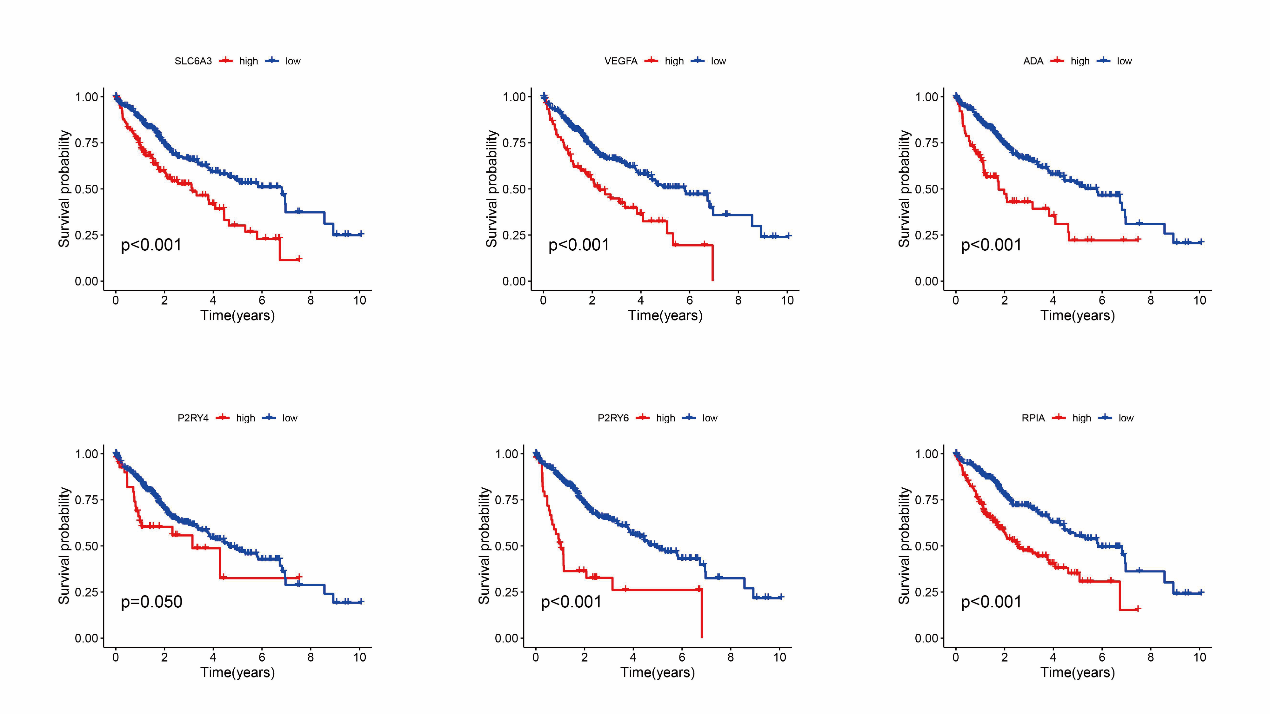
 **Online Resource 7.** The Kaplan-Meier survival analysis was performed on genes involved in the construction of the risk score related to adenosine metabolism (ADA, P2RY4, P2RY6, RPIA, SLC6A3, and VEGFA).
